# Analysis of Interaction Network Between Host Protein and M Protein of Swine Acute Diarrhea Syndrome Coronavirus

**DOI:** 10.3389/fmicb.2022.858460

**Published:** 2022-04-08

**Authors:** Jingya Xu, Ze Cao, Chihai Ji, Ling Zhou, Xiaoling Yan, Yuan Sun, Jingyun Ma

**Affiliations:** ^1^Guangdong Provincial Key Lab of Agro-Animal Genomics and Molecular Breeding, College of Animal Science, South China Agricultural University, Guangzhou, China; ^2^Guangdong Laboratory for Lingnan Modern Agriculture, Guangzhou, China

**Keywords:** swine acute diarrhea syndrome coronavirus (SADS-CoV), membrane (M) protein, protein interaction network, RPL18, RALY, RHOA

## Abstract

Swine acute diarrhea syndrome coronavirus (SADS-CoV) is an enterovirus that can cause acute diarrhea and death in piglets and cause serious economic losses to the pig industry. SADS-CoV membrane (M) protein mainly plays a key role in biological processes, such as virus assembly, budding, and host innate immune regulation. Understanding the interaction between M protein and host proteins is very important to define the molecular mechanism of cells at the protein level and to understand specific cellular physiological pathways. In this study, 289 host proteins interacting with M protein were identified by glutathione-S-transferase (GST) pull-down combined with liquid chromatography-mass spectrometry (LC-MS/MS), and the protein-protein interaction (PPI) network was established by Gene Ontology (GO) terms and Kyoto Encyclopedia of Gene and Genomes (KEGG) pathways analysis. Results showed that SADS-CoV M protein was mainly associated with the host metabolism, signal transduction, and innate immunity. The Co-Immunoprecipitation (CO-IP) validation results of six randomly selected proteins, namely, Rab11b, voltage-dependent anion-selective channel 1 (VDAC1), Ribosomal Protein L18 (RPL18), RALY, Ras Homolog Family Member A (RHOA), and Annexin A2 (ANXA2), were consistent with LC-MS results. In addition, overexpression of RPL18 and PHOA significantly promoted SADS-CoV replication, while overexpression of RALY antagonized viral replication. This work will help to clarify the function of SADS-CoV M protein in the life cycle of SADS-CoV.

## Introduction

Swine acute diarrhea syndrome coronavirus (SADS-CoV) is a newly discovered coronavirus (Gong et al., 2017; Pan et al., 2017; Zhou et al., 2018b). It is an enveloped positive single-stranded sense RNA virus, which belongs to the Alphacoronavirus genus of the Coronaviridae family and is considered to be a novel bat coronavirus that is highly similar to HKU2 (Zhou et al., 2019). SADS-CoV can cause severe acute diarrhea and weight loss to piglets, resulting in serious economic losses (Zhou et al., 2019).

The genome of SADS-CoV is about 27 Kb and consists of nine open reading frames (ORF), including ORF1A, ORF1B, spike (S), envelope (E), membrane (M), nucleocapsid (N), and three auxiliary proteins, namely, NS3a, NS7a, and NS7b (Zhou et al., 2021). The membrane (M) protein is the most abundant structural protein in virions that includes a short N-terminal outer domain, three transmembrane domains, and a C-terminal inner region (Wang et al., 2020b). This protein is mainly involved in the assembly and release of the virus and is also involved in the innate immune response of the host. For example, some studies have shown that severe acute respiratory syndrome coronavirus 2 (SARS-CoV-2) M protein could inhibit the production of type I and type III interferon induced by the cytoplasmic double-stranded RNA (dsRNA) sensing pathway that is mediated by retinoic acid-inducible gene I (RIG-I) signal transduction (Zheng et al., 2020) and could manipulate autophagy (Koepke et al., 2021). In the process of viral invading, to exert its function, viral proteins interact with host proteins to form complexes and high-order network structures that can control cell physiology. To investigate interactions between viruses and hosts, proteins may provide useful evidence for hijacking mechanisms by which viruses use to gain benefits. However, the role of SADS-CoV M protein in this process is not clear.

In this study, we have identified 289 host proteins that interact with SADS-CoV M protein by glutathione-S-transferase (GST) pull-down and liquid chromatography-mass spectrometry (LC-MS/MS). Subsequently, the bioinformatics analysis of these proteins was carried out, and the protein-protein interaction (PPI) network was constructed, which would be helpful to understand the interaction between M protein and host proteins and to uncover the pathogenesis and immune escape mechanisms used by SADS-CoV.

## Materials and Methods

### Cell, Virus, and Plasmid

IPI-2I cells and Vero cells were kept in our laboratory and cultured in Dulbecco's Modified Eagle Medium (DMEM, HyClone) that was supplemented with 10% fetal bovine serum (FBS, HyClone) at 37°C within 5% CO_2_ incubator. The SADS-CoV/CN/GDWT/2017 strain (GenBank accession number MG557844) was isolated from diarrhea samples of suckling piglets in a commercial pig farm in Guangdong Province in April 2017 and was kept in our laboratory. Vero cells were infected with the dose of 1 multiplicity of infection (MOI), and the samples were collected for Median Tissue Culture Infectious Dose (TCID_50_) determination at 36 h. TCID_50_ was calculated by the Reed-Muench method (Huan et al., 2022).

Membrane protein, Rab11A, voltage-dependent anion-selective channel 1 (VDAC1), Ribosomal Protein L18 (RPL18), RALY, Ras Homolog Family Member A (RHOA), and Annexin A2 (ANXA2) expression plasmids containing tags were generated using the homologous recombinant method. All the primer sequences in this study would be available upon request. M gene was amplified by RT-PCR using SADS-CoV/CN/GDWT/2017 RNA as template and cloned into vector pCAGGS-HA and pGEX-6p-1 with homologous recombinant reagent (one-step cloning kit, Vazyme, China) to form the recombinant plasmid pCAGGS-M-HA and pGEX-6p-1-M. The RT-PCR using full-genome RNA of IPI-2I cells as template was employed to amplify the Rab11A/VDAC1/RPL18/RALY/RHOA/ANXA2-Flag genes, which were cloned into vector pCAGGS with, respectively, homologous recombination reagent to obtain the recombinant plasmids pCAGGS-Rab11A/VDAC1/RPL18/RALY/RHOA/ANXA2-Flag. All plasmids were verified by sequencing.

### Antibodies and Reagents

Anti-GAPDH mouse monoclonal antibody (mAb), anti-Flag mAb, and anti-HA mAb were obtained from Proteintech (Wuhan, China). Glutathione agarose beads for GST pull-down were purchased from BEAVER (Suzhou, China), and Lipofectamine 3000 was purchased from Life Technologies (Invitrogen, USA). Cell NP-40 lysis buffer [50 mM Tris (pH 7.4), 150 mM NaCl, 1% NP-40] was purchased from Beyotime (P0013F; Shanghai, China). Horseradish peroxidase (HRP)-labeled goat anti-mouse and anti-rabbit immunoglobulin G (IgG) were purchased from KPL (Milford, MA, USA). Mouse mAb to N of SADS-CoV was produced by our laboratory.

### Sodium Dodecyl Sulfate-Polyacrylamide Gel Electrophoresis and Immunoblotting

For the Western blotting, beads were lysed in lysis buffer after Co-Immunoprecipitation (Co-IP) or GST pull-down assays, and proteins were separated by standard SDS-PAGE, transferred to polyvinylidene difluoride (PVDF) membranes (GE Healthcare) followed by blocking in PBS containing 5% skim milk and 0.05% Tween 20, and washed with PBS containing 0.05% Tween 20. The membranes were incubated with primary antibody overnight at 4°C. After three to five washes with PBS containing 0.1% Tween 20, the membranes were incubated with HRP-labeled secondary antibody at room temperature for 1.0 h. Protein blots were detected using enhanced chemiluminescence (ECL) detection system and an Azure c600 visible fluorescent Western blot imaging system (Azure Biosystems, USA).

### RNA Extraction and Quantitative Real-Time RT-qPCR

TRIzol reagents (Invitrogen, USA) were used to extract total RNA from the treated samples, and a PrimeScript™ RT Kit (Takara, Biotechnology, Dalian, China) was used. Then the cDNA was analyzed by the method established in our laboratory (Zhou et al., 2018a). Each sample was repeated 3 times.

### Preparation of GST Fusion Proteins

The pGEX-6p-1-M plasmids containing coding sequences for full-length SADS-CoV M were transformed into *Escherichia coli* (*E. coli*) strain BL21. Single colonies were grown on Luria-Bertani (LB) plate overnight at 37°C and selected for enlarging cultivation in LB medium at 37°C until the optical density (600 Nm) reached 0.6, and then 0.1 mM isopropyl-β-D-thiogalactoside (IPTG) was added for induction at 20°C overnight. Proteins were obtained by sonication of the cells and purified by agarose affinity chromatography and then solubilized to Glutathione agarose beads according to the instructions provided by the manufacturer.

### GST Pull-Down Assays and CO-IP Analysis

Membrane protein that was dissolved in glutathione-agarose beads was incubated with IPI-2I cell lysate overnight at 4°C, and glutathione-agarose beads were used as the negative control. Subsequently, the beads were washed with the incubation buffer to remove the unbound proteins, the magnetic beads were collected, and the 1× SDS-PAGE sample buffer was added. Then SDS-PAGE was performed and analyzed by Coomassie Brilliant Blue staining.

IPI-2I and Vero cells grown in 60-mm dishes were transfected with expression plasmids and the whole-cell lysates were collected by cell lysis buffer for Western blot and immunoprecipitation. That is, samples were immunoprecipitated with the protein A/G magnetic beads and incubated with the antibody for 4 h at 4°C or immunoprecipitated with Anti-Flag Affinity Gel overnight at 4°C. After three times of washing by phosphate-buffered saline and Tween 20 (PBST), the Anti-Flag Affinity Gel or protein A/G magnetic beads were added with 50 μl 1× SDS-PAGE sample loading buffer and boiled for 5 min at 100°C. The levels of protein expression after immunoprecipitation were then analyzed by Western blot.

### LC-MS/MS

The cut gel was destained by 50% acetonitrile (ACN) in 50 mM triethylammonium bicarbonate (TEAB) and dehydrated upon washing with 100% ACN till the gel turned white. Gel were treated with 1,000 μl 10 mM dithiothreitol (DTT) for 40 min at 56°C and subsequently alkylated with 1,000 μl 50 mM Iodoacetamide (IAM) for 30 min in the dark. The gel was then washed by detained buffer and treated with ACN as above.

In total, 10–20 μl 10 ng/μl trypsin was added into gel and incubation for 30 min on ice, then made up to 100 μl with 100 mM tetraethylammonium bromide (TEAB), proteins were digested overnight at 37°C. After centrifugation at a low speed, the supernatant was collected and the remaining peptides were extracted in 100 μl of 0.1% formic acid (FA). Then, combined the two supernatant and centrifuged at 12,000 *g* for 5 min at room temperature. The supernatant was slowly loaded to the C18 desalting column, washed with 1 ml of washing solution (0.1% formic acid, 4% acetonitrile) 3 times, and then eluted twice by 0.4 ml of elution buffer (0.1% formic acid, 75% acetonitrile). The eluents were combined and lyophilized.

The lyophilized powder was dissolved in 10 μl of solution A (100% water, 0.1% formic acid), centrifuged at 15,000 rpm for 20 min at 4°C, and 1 μg of the sample was injected into a home-made C18 Nano-Trap column (2 cm × 75 μm, 3 μm). Peptides were separated in a home-made analytical column (15 cm × 150 μm, 1.9 μm), using linear gradient elution. The separated peptides were analyzed by Q Exactive HF-X Mass Spectrometer (Thermo Fisher). The top 40 precursors of the highest abundant in the full scan were selected and fragmented by higher-energy collisional dissociation (HCD) and analyzed in MS/MS, where the resolution was 15,000 (at m/z 200), the automatic gain control (AGC) target value was 1 × 10^5^, the maximum ion injection time was 45 ms, normalized collision energy was set as 27%, an intensity threshold was 2.2 × 10^4^, and the dynamic exclusion parameter was 20 s.

### Construction and Analysis of the PPI Network

Based on all datasets, the SADS-CoV M protein-host protein interaction network was generated using Cytoscape v.3.8.2. To analyze the interaction between host and proteins, a STRING database was used. Topological parameters and central measures of the network were calculated using a network analyzer tool in Cytoscape v.3.8.2. Pig PPI analysis was also performed using the STRING database.

### Gene Ontology and Kyoto Encyclopedia of Gene and Genomes Pathway Analyses

Gene Ontology analysis was conducted using the Interproscan-5 program against the non-redundant protein database (such as Pfam, PRINTS, ProDom, SMART, PROSITE, and PANTHER), and the database KEGG were used to analyze the protein family and pathway.

### Statistical Analysis

All experiments were independently repeated at least three times, and all data are presented as means ± SD. The significance of the experimental results was analyzed using GraphPad Prism v.8 (San Diego, CA, USA). The experimental data with *p* < 0.05 are considered to be statistically significant. *p* between 0.05 and 0.01 is marked as “^*^” and *p* < 0.01 is marked as “^+^”.

## Results

### Identification of Host Factors Interacting With SADS-CoV M in IPI-2I Cells

**Figure 1:**
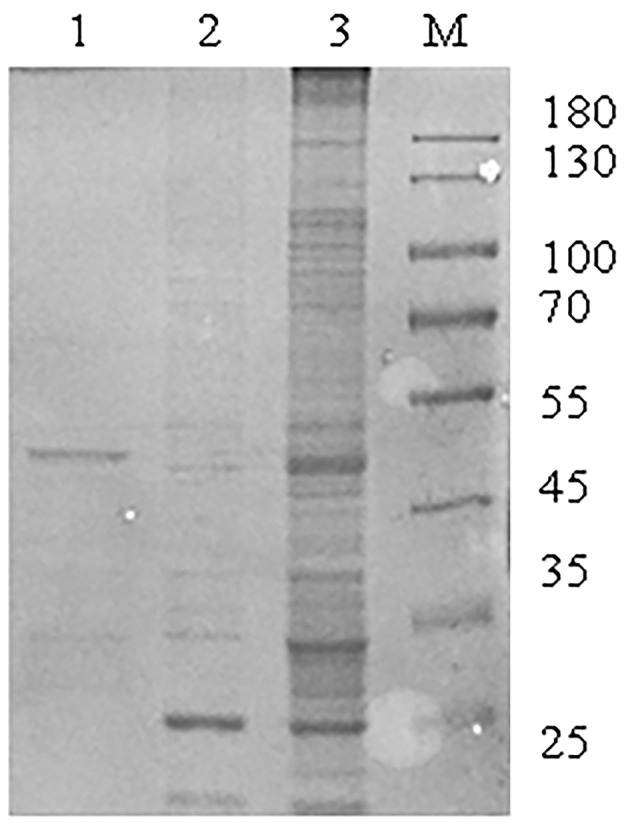
Identification of interaction factors between Swine acute diarrhea syndrome coronavirus (SADS-CoV) M protein and host. A large number of glutathione-S-transferase (GST)-M recombinant proteins were expressed and purified by magnetic beads with GST fusion protein for purification and GST pull-down experiments. Then sodium dodecyl sulfate-polyacrylamide gel electrophoresis (SDS-PAGE) was used for isolation and identification. M: page ruler protein marker; Lane 1: purified GST-M fusion protein elution solution; 2: GST protein pull-down result; 3: GST-M protein pull-down result.

To investigate host proteins that interact with SADS-CoV M protein, the immunoprecipitation analysis using GST pull-down was performed with cell lysates and purified M protein. The Coomassie blue staining was used to observe host proteins that were bounded with SADS-CoV M protein (Figure 1). As a negative control, cell lysates were combined with magnetic beads to eliminate non-specific interactions. Different bands could be observed when compared to the negative control. Subsequently, LC-MS/MS was performed for identification. Results showed that a total of 289 cell proteins interacting with SADS-CoV M protein were identified.

### Equations Construction and Analysis of the PPI Network

**Figure 2:**
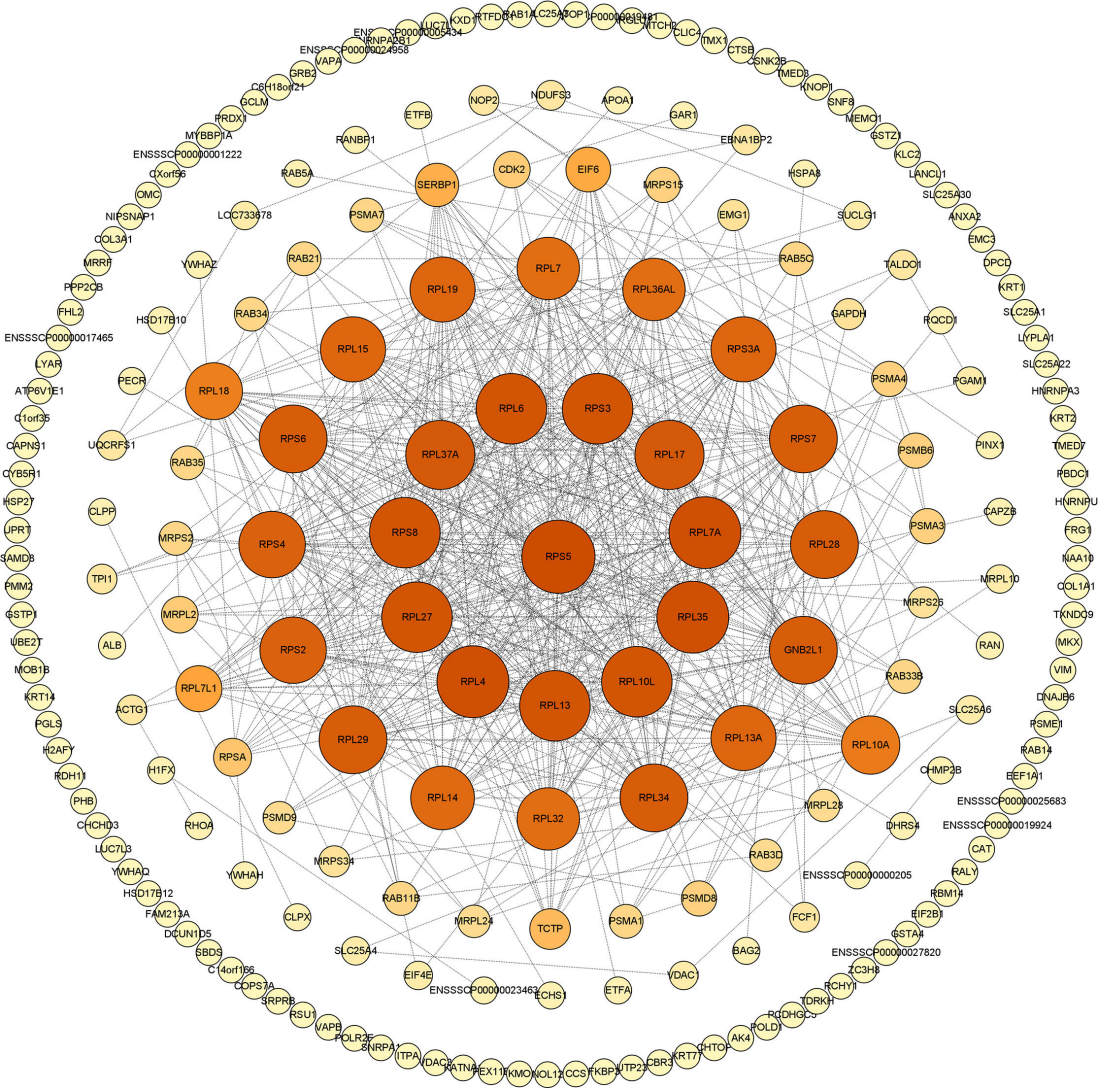
The PPI network generated in this study. Construction and analysis of the protein-protein interaction (PPI) network using the STRING database. The network of swine acute diarrhea syndrome coronavirus (SADS-COV) M-interacting proteins interacting was constructed and plotted using the network analyzer tool, Cytoscape v.3.8.2. The nodes are identified host proteins, expressed as their respective NCBI gene names. Edges represent the interaction between nodes. The higher the degree, the larger the node and the darker the color.

*In vivo*, proteins usually play a series of biological roles through complex interactions. Therefore, the mapping of PPIs and their interaction networks (PPI) is very important for understanding cellular tissues, biological processes, and functions. In this study, we used the STRING database and Cytoscape v.3.8.2. software to build and draw the PPI network. Results of the network topology analysis were as follows: network density (0.330), network heterogeneity (0.713), and average number of neighbors (18.491). The number of sides of the observed network (1,527) is significantly higher than expected (652), indicating that the number of interactions is higher than expected (Figure 2).

### GO Annotation and Analysis

**Figure 3:**
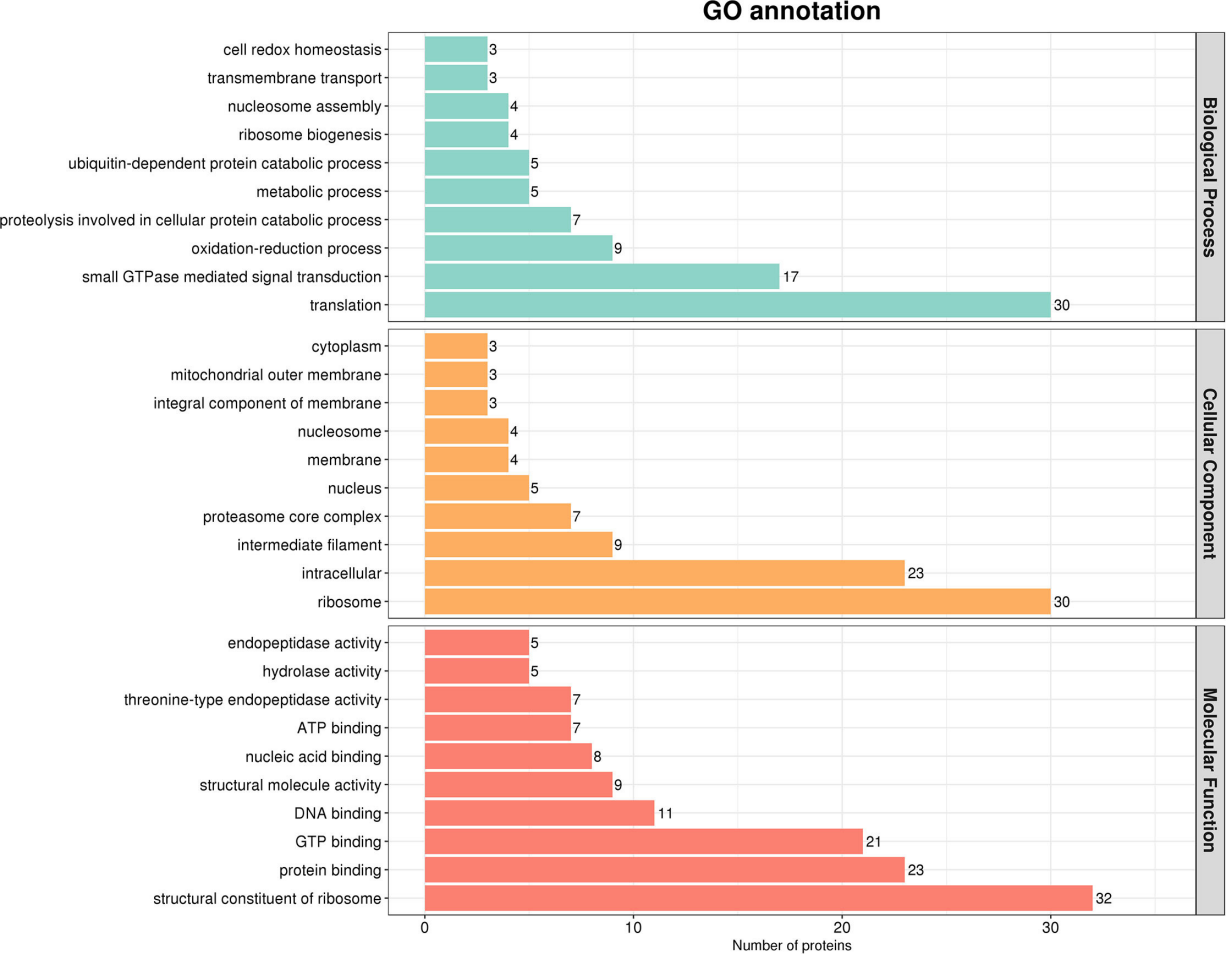
Gene ontology (GO) analysis based on the interaction between cell protein and M. Using Cytoscape v.3.8.2 and ClueGO software plug-in, the GO distributions of all proteins were divided into three types. The y was shown for significantly enriched terms based on biological process, molecular function (MF), and cellular component at *p* < 0.05.

After that, we used Interproscan-5 to annotate the protein GO according to the identification results. As shown in Figure 3, gene ontological enrichment analysis consists of three categories, namely, biological processes, molecular functions, and cellular components. The results showed that SADS-CoV M protein mainly affected the biological processes, such as translation, small GTPase-mediated signal transduction, and oxidation-reduction process, in the host. Moreover, it mainly holds ribosome, intracellular, intermediate filaments, and other cellular components. In addition, a large number of targeted molecular functions, such as structural constituent of ribosome, protein binding, and guanosine triphosphate (GTP) binding. Generally speaking, the GO annotation and analysis of the target protein inferred that SADS-CoV M protein mainly functions in the cytoplasm, which may affect the host metabolism, protein translation innate immunity, etc.

### KEGG Pathway Enrichment Analysis

**Figure 4:**
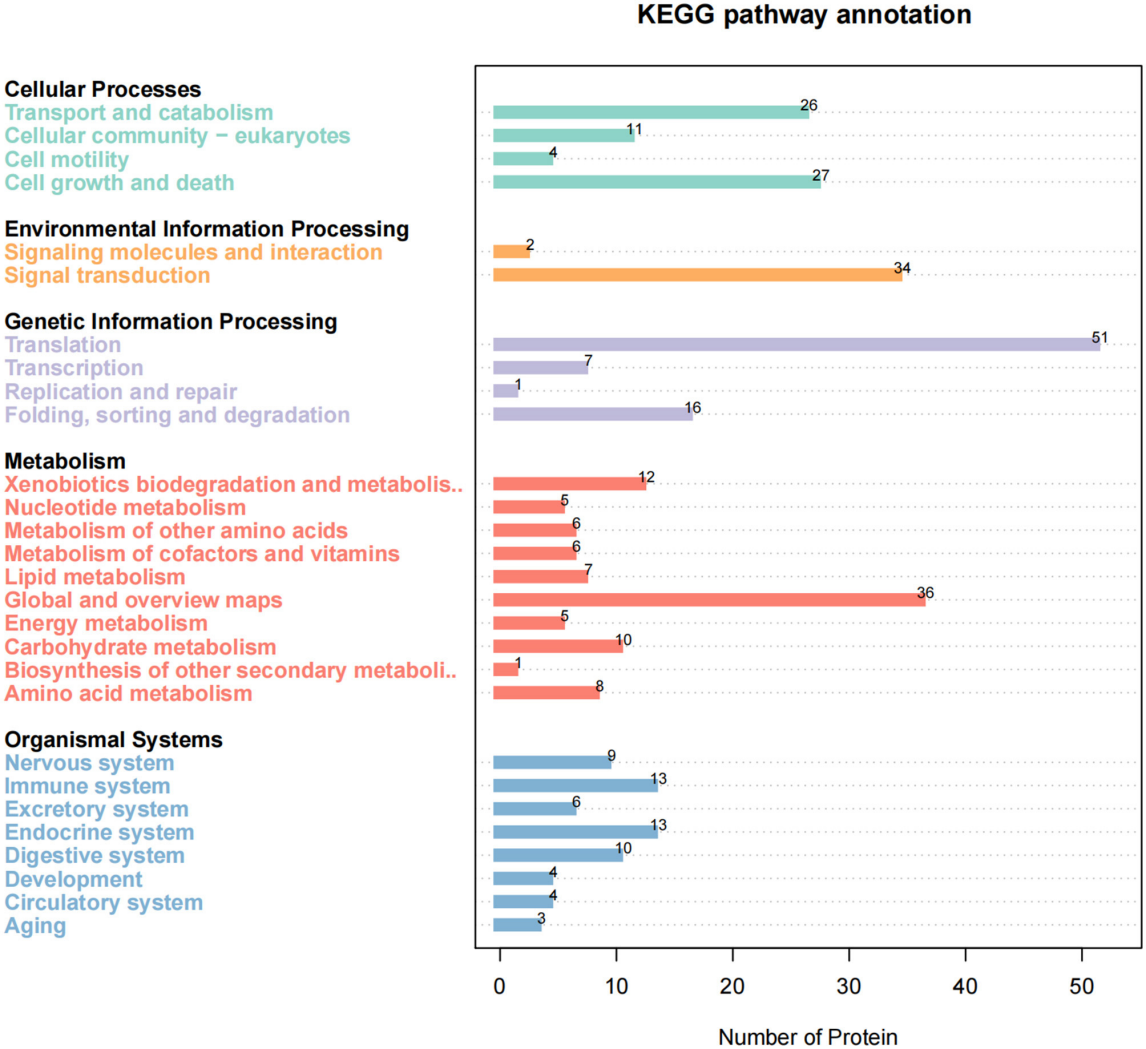
KEGG pathway enrichment analysis. The enriched pathways targeted by Swine acute diarrhea syndrome coronavirus (SADS-COV)-M-interacting proteins were analyzed using the Kyoto Encyclopedia of Gene and Genomes (KEGG) functional annotation pathway database. The terms that were significantly enriched (*p* < 0.05) were shown.

To further reveal the cellular pathway of host protein metabolism and signal transduction targeted by SADS-CoV M protein, we used KEGG to analyze the pathway enrichment. The results showed that the target proteins were mainly involved in the ribosome and its biosynthesis, multiple metabolic pathways, PI3K-ATK signal pathways, proteasome, and necrotizing apoptosis. Interestingly, these proteins are also associated with tight junction proteins, endocytosis, Ca^+^ signaling pathways, etc. Enrichment analysis showed that these proteins were mainly related to signal transduction, transport and catabolism, cell life cycle, and other functions (Figure 4).

### Verify the Interaction Between Host Protein and SADS-CoV M Protein

**Figure 5:**
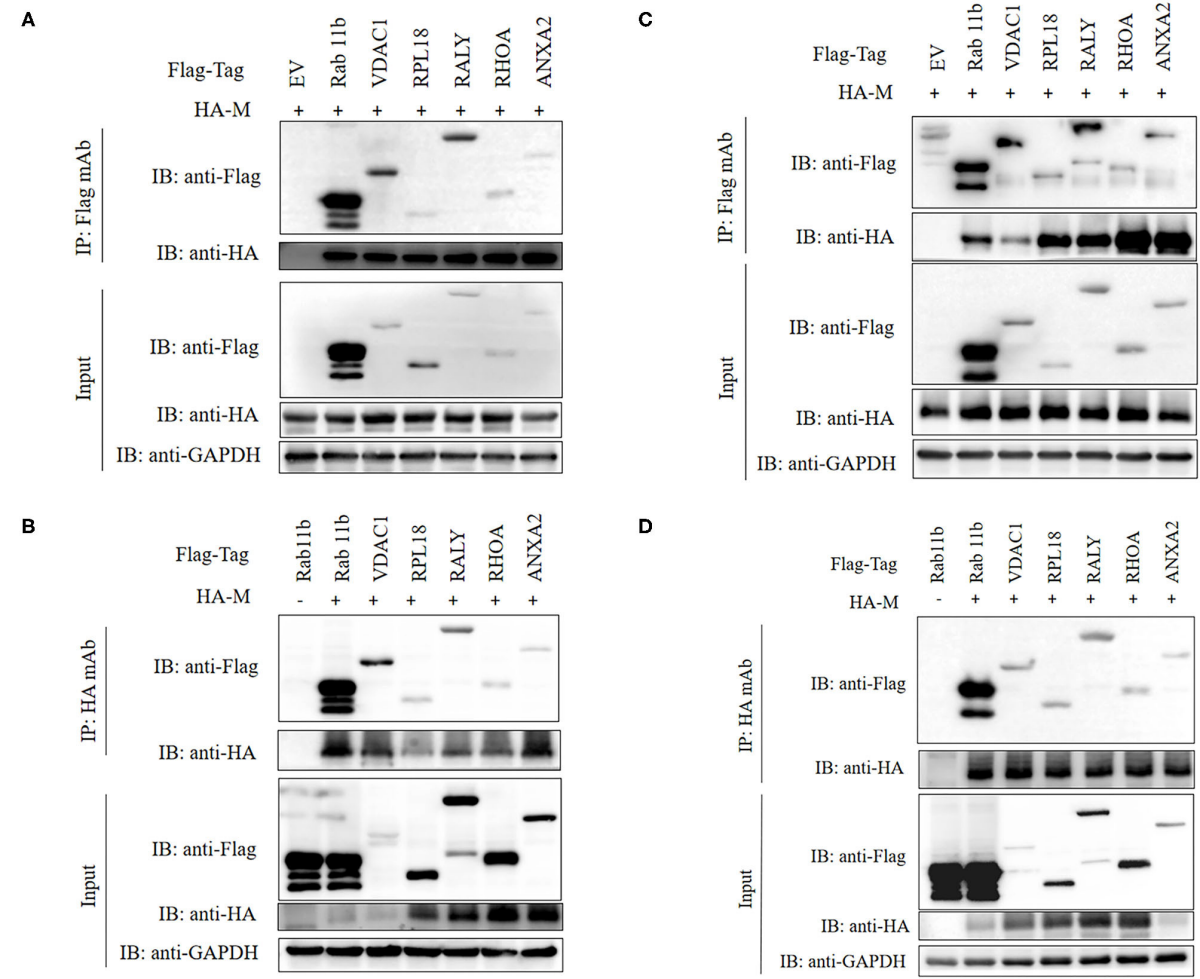
Validation of M-host protein interactions. IPI-2I **(A,B)** and Vero **(C,D)** cells were co-transfected with plasmids expressing Flag-Rab11b, Flag-VDAC1, Flag-RPL18, Flag-RALY, Flag-RHOA, or Flag-ANXA2 and plasmids expressing HA-M, respectively. Among them, HA-M or Flag-Rab11b co-transfected with empty vector were served as negative controls, respectively. The cell lysates were immunoprecipitated with FLAG **(A,C)** or HA **(B,D)** beads, anti-Flag, or anti-HA mAbs, separated by sodium dodecyl sulfate-polyacrylamide gel electrophoresis (SDS-PAGE), Western-blotted and detected with corresponding antibodies, respectively. GAPDH was served as an internal loading control.

To verify the PPI obtained by LC-MS/MS, we carried out CO-IP experiments *in vitro*. Six different host proteins were randomly selected from cellular proteins that interact with SADS-CoV M protein. To eliminate the effect of labeling on the authenticity of PPI, we selected HA-M and empty vectors, PCDNA3.1 (+)-Flag, Flag-Rab11b, Flag-VDAC1, Flag-RPL18, Flag-RALY, Flag-RHOA, and Flag-ANXA2, to co-transfect IPI-2I cells and Vero cells and then, respectively, immunoprecipitated with HA mAb and Flag mAb. The results showed that M had specific interaction with Rab11b, VDAC1, RPL18, RALY, RHOA, and ANXA2 (Figure 5).

### Overexpression of RPL18, RALY, and RHOA Affected Virus Replication

**Figure 6:**
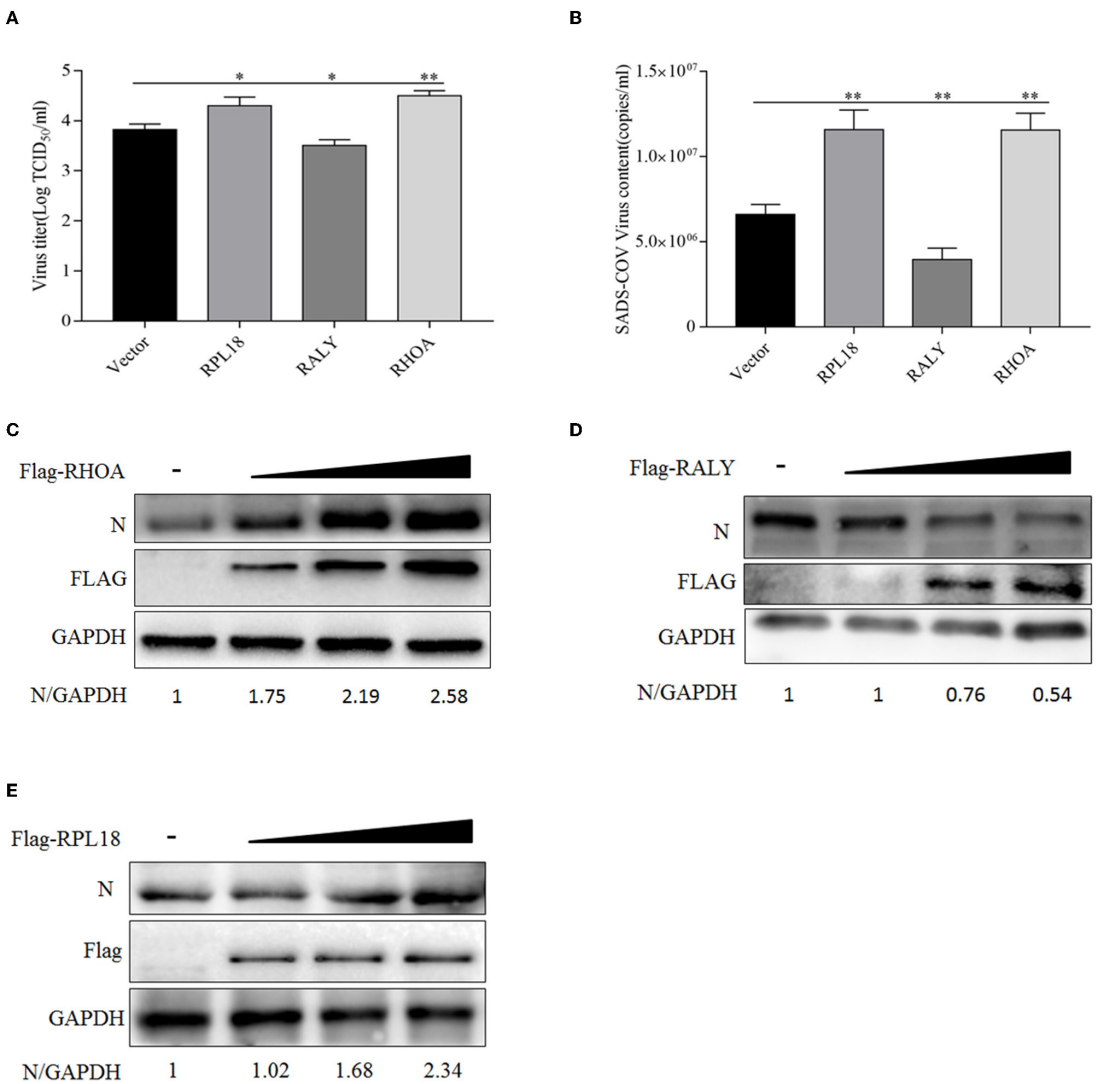
Overexpression of host proteins RPL18, RALY, and RHOA affects swine acute diarrhea syndrome coronavirus (SADS-COV) replication. **(A)** When the Vero cells were laid on 12-well plates, and the confluence degree reached 80%, empty vector (4 μg/well), Flag-RPL18 (4 μg/well), Flag-RALY (4 μg/well), and Flag-RHOA (4 μg/well) were transfected into the cells. At 24 h after transfection, 1 multiplicity of infection (MOI) dose of SADS-COV was added to the cell. Viral titers were detected by TCID50 at 36 hpi. **(B)** Sample treatment as described in **(A)**. After 36 hpi, the samples were collected by RNAiso Plus, and the total RNA was extracted. The mRNA expression level of SADS-CoV N gene was evaluated by real-time RT-qPCR. Flag-RHOA **(C)**, Flag-RALY **(D)**, and Flag-RPL18 **(E)** were transfected into Vero cells at different doses (1, 2l, and 4 μg/well) with empty vector, and the rest were treated as described in **(A)**. N protein expression was detected by Western blotting at 36 hpi. GAPDH was used as an internal loading control. The band densitometry was analyzed by Image J software. All data are presented as means ± SD and were analyzed by GraphPad Prism software (GraphPad Software, San Diego, CA, USA) using a *t*-test (*0.05 < *p* < 0.01, **p < 0.01).

Among those six randomly selected proteins, the functions of RPL18, RALY, and RHOA have attracted our attention. To determine the effect of RPL18, RALY, and RHOA on SADS-COV replication, PCDNA3.1 (+)-FLAG, Flag-RHOA, Flag-RALY, or Flag- RPL18 were transfected into Vero cells, respectively. Twenty-four hours post-inoculation (hpi) after transfection, 1 MOI dose of SADS-CoV was added to the cells. The infection rate of the virus in the cells expressing recombinant protein was observed at 36 hpi after infection. Evaluation of virus replication was based on SADS-COV N protein. At the same time, samples were collected to determine the virus titer. The results showed that the titer of SADS-CoV virus in the samples overexpressing RHOA and RPL18 recombinant plasmids was significantly higher than that in the control group, and the mRNA and protein levels of SADS-CoV N protein were also significantly upregulated. On the contrary, the virus titer in the samples overexpressing RALY recombinant plasmid was significantly lower than that in the control group, and the mRNA and protein levels of N protein were the same as those in the control group. In addition, from the protein level, it can be seen that the regulation of virus replication by the above three proteins is dose-dependent (Figure 6).

## Discussion

In recent years, the continuous emergence of highly pathogenic viruses, such as SARS-CoV-2, severe acute respiratory syndrome coronavirus (SARS-CoV), and Middle East respiratory syndrome-related coronavirus (MERS-CoV), has a significant impact on public health, society, and the global economy (Terrier et al., 2021; Wang et al., 2021). SADS-CoV is highly similar to HKU2. Studies have shown that it shows a wide range of species tendency and replicates effectively in human cells (Yang et al., 2019; Edwards et al., 2020). However, at present, we do not have a deep understanding of this virus. Considering the characteristics of rapid mutation and interspecies transmission risk of coronavirus, we urgently need to understand the molecular mechanism of SADS-CoV replication and pathogenesis. In fact, viruses have evolved a variety of mechanisms to hijack host proteins for self-replication and evade the host's defense system and innate immunity (Fung and Liu, 2019; Perrin-Cocon et al., 2020). Exploring the interaction between SADS-CoV M protein and host protein will help us to understand the life cycle of the virus and lay the foundation for the development of vaccines and antiviral drugs.

In this study, we used GST pull-down combined with LC-MS/MS technology to explore host proteins interacting with SADS-CoV M protein in the IPI-2I cell line for the first time. We screened and identified 289 host proteins that may interact with SADS-CoV M protein and drew the PPI network map. Through STRING analysis, the expected edge number is 652, and the actual observed edge number is 1,527, indicating that there are more PPIs than expected. It is inferred that these 289 host proteins may form regulatory networks with more complex relationships, or they may function mainly in the form of protein complexes. Moreover, the viral invasion may destroy the original regulatory network, thus forming another regulatory mechanism that is more conducive to virus replication. In addition, we observed that host proteins that are targeted by M protein form a PPI network centered on ribosomal proteins. As we all know, the function of the ribosome is mainly responsible for translation regulation within the host, and many researchers have found that ribosomes play an indispensable role in the life cycle of viruses (Li, 2019; Miller et al., 2021). Therefore, we speculated that SADS-CoV M protein may create conditions for viral translation by targeting ribosomal proteins in the host, which deserve further studies. Notably, GNB2L1 protein has appeared in the central area of the PPI network. Studies have shown that GNB2L1 is related to RNA binding and signal transduction in the host (Buoso et al., 2017). This suggests that M protein may mediate a series of signal transduction through the interaction with GNB2L1 to provide support for the translation of viral RNA. In a word, the PPI network of SADS-CoV M protein indicates that M protein is closely related to the host translation system, which further proves that M protein plays an indispensable key role in the life cycle of SADS-CoV.

Our results based on GO analysis showed that the host proteins interacting with SADS-COV M protein were mainly located in the cytoplasm, ribosome, and many kinds of biomembrane. Therefore, M protein may mediate the transport, assembly, and release of virus protein mainly by targeting the M protein in the host. In addition, it can be seen that M protein is also related to protein degradation in the host. Studies have shown that SARS-CoV-2 M protein can mediate the degradation of mitochondria (Hui et al., 2021). Whether there is the same regulatory mechanism of SADS-CoV M protein remains to be confirmed by further studies. Furthermore, the analysis of the KEGG pathway showed that a large number of host proteins interacting with M protein are mainly involved in the ribosomal pathway, apoptosis, autophagy, cell cycle, adenosine monophosphate-activated protein kinase (AMPK) signal pathway and multiple metabolic pathways, PI3K-AKT signal pathway, exocytosis, proteasome, splice body signal pathway, and other important signal pathways in the host body. It is known that the AMPK signal pathway is linked to autophagy and apoptosis (Wang et al., 2022), so there is a possibility that M protein may regulate autophagy and apoptosis by targeting key proteins in the AMPK signal pathway. Interestingly, some host proteins are associated with other viruses, such as Epstein-Barr (EB) virus, hepatitis B, human papillomavirus, hepatitis C, and herpes simplex virus. We speculate that there may be similar regulatory mechanisms between SADS-CoV and other pathogens in the process of infection, which suggests that we can focus on the research progress of these pathogens in related research. Studies have shown that Porcine epidemic diarrhea virus (PEDV) M proteins are involved in the regulation of a variety of signal pathways, which are related to Rab family members, translation factors, and Rho family (Wang et al., 2020a). The prediction of M protein function in this study is consistent. This suggests that the M proteins of these two viruses may be involved in regulating the same biological process, but whether the target proteins and their mechanisms are the same remains to be verified.

In the current study, we randomly selected six host proteins, namely, Rab11b, VDAC1, RPL18, RALY, RHOA, and ANXA2, and verified their interaction with SADS-CoV M proteins by Co-IP experiments. The results showed that the results of the LC-MS/MS analysis were reliable. Among them, we focus on the role of ribosomal proteins, RNA-binding proteins, and Ras superfamily in virus replication.

At present, more and more researchers pay attention to the relationship between the ribosome and the virus. Ribosomal proteins play an important role in the life cycle of viruses, such as promoting virus translation, participating in virus assembly and replication, and acting as virus receptors (Li, 2019; Dong et al., 2021; Miller et al., 2021). RPL18 belongs to the ribosomal 60s subunit, and some studies have shown that it is involved in ribosome assembly. It is reported that PRL18 interacts with virus proteins of Newcastle disease virus (NDV), rice stripe virus (RSV), and Ebola virus and uses this interaction to affect virus replication (Spurgers et al., 2010; Li et al., 2018; Duan et al., 2021). RPL18 of Arabidopsis thaliana interacted with P6 of cauliflower mosaic virus (CaMV) in a complex consisting of several ribosomal proteins and the translation initiation factor eIF3, which was required for translational transactivation of CaMV (Leh et al., 2000; Bureau et al., 2004). RPL18 forms complexes with infectious bursal disease virus (IBDV) VP3 and PKR to regulate virus replication by regulating the expression of type I interferon (Wang et al., 2018). In our study, we observed that overexpression of RPL18 on Vero cells could promote virus replication. It is inferred that in the process of SADS-CoV replication, M protein may complete its own biological processes, such as translation or translation co-folding by hijacking RPL18, or regulate host immune response by regulating RPL18, thus affecting virus replication. However, the principle and mechanism of its interaction need to be demonstrated in future work. To summarize, RPL18 has the potential to become a new antiviral target of SADS-CoV.

In this study, we also confirmed the interaction between RALY and SADS-CoV M proteins. Moreover, when compared with the negative control, the increase of RALY expression level significantly decreased the production of SADS-CoV, indicating that this factor played a negative regulatory role in virus replication. RALY is a member of the nuclear heterogeneous ribonucleoprotein family (Heterogeneous Nuclear Ribonucleoprotein, hnRNP). As an RNA-binding protein, it plays a role in mRNA splicing and metabolism and is related to the cell cycle (Rossi et al., 2017). Some studies have shown that the ubiquitination of RALY and hnRNP-C mediated by Adenovirus (AD) removes the restriction on viral RNA processing and reveals the unexpected role of non-degradable ubiquitination in the manipulation of cellular processes during virus infection (Herrmann et al., 2020). More studies are needed to reveal the role of RALY in SADS-CoV infection.

Ras Homolog Family Member A, a member of the RAS superfamily, plays an important role in cytoskeleton remodeling, cell cycle progression, cell migration, and gene expression by interacting with a variety of regulatory proteins and effectors (Chun, 2021; DeOre et al., 2021). Studies have shown that porcine sapovirus and rotavirus regulate tight junctions through the RHOA signal pathway and then affect virus replication (Soliman et al., 2018; Sharif et al., 2021). Human parainfluenza virus type 2, IBDV, and pseudorabies virus rely on RHOA to induce actin to affect virus replication (Jacob et al., 2015; Ye et al., 2017; Ohta et al., 2018). In this study, we found that the upregulation of RHOA expression could promote virus replication. Future work will focus on whether M protein and RHOA crosstalk can mediate cytoskeleton remodeling, thus affecting virus replication, assembly, and release, as well as the roles they play in this process.

In a word, 289 host proteins that interact with SADS-CoV M protein were identified by combining GST pull-down and LC-MS/MS. These proteins are involved in a variety of signal pathways, such as immune response, apoptosis, ribosome, and its biosynthesis, and are involved in a wide variety of biological processes. Six host proteins were randomly selected to verify the gyration, which proved the reliability of the MS results. Among them, we have verified that the changes in the expression of RPL18, RALY, and RHOA will affect the replication of the virus, and the specific mechanism needs to be further studied. Based on the interaction between M protein and multiple virus proteins, it is more important to explore the interaction between M protein and host. This work can provide guidance for the development of antiviral drugs with a view to blocking viral replication at different stages of the life cycle of the virus.

## Data Availability Statement

The original contributions presented in the study are included in the article/supplementary material, further inquiries can be directed to the corresponding authors.

## Author Contributions

JM and YS conceived and designed the study and critically revised the manuscript. JX wrote the manuscript, performed the experiments, and conducted data analysis. ZC, CJ, LZ, and XY helped in experimental implementation. All authors contributed to the article and approved the submitted version.

## Funding

This work was financially supported by the Guangdong Major Project of Basic and Applied Basic Research (No. 2020B0301030007), the Science and Technology Program of Guangzhou City of China (201904010433), and Natural Science Foundation of Guangdong Province (2020A1515010220 and 2020A1515010295).

## Conflict of Interest

The authors declare that the research was conducted in the absence of any commercial or financial relationships that could be construed as a potential conflict of interest.

## Publisher's Note

All claims expressed in this article are solely those of the authors and do not necessarily represent those of their affiliated organizations, or those of the publisher, the editors and the reviewers. Any product that may be evaluated in this article, or claim that may be made by its manufacturer, is not guaranteed or endorsed by the publisher.
